# Rab7B/42 Is Functionally Involved in Protein Degradation on Melanosomes in Keratinocytes

**DOI:** 10.1247/csf.19039

**Published:** 2020-02-07

**Authors:** Soujiro Marubashi, Mitsunori Fukuda

**Affiliations:** 1 Laboratory of Membrane Trafficking Mechanisms, Department of Integrative Life Sciences, Graduate School of Life Sciences, Tohoku University, Aobayama, Aoba-ku, Sendai, Miyagi 980-8578, Japan

**Keywords:** degradation, keratinocytes, melanocytes, melanosome, Rab small GTPase

## Abstract

Keratinocytes uptake melanosomes from melanocytes and retain them in the perinuclear region, where they form melanin caps. Although these processes are crucial to protecting nuclear DNA against ultraviolet injury, the molecular basis of melanosome uptake and decomposition in keratinocytes is poorly understood. One of the major reasons for its being poorly understood is the lack of a specific marker protein that can be used to visualize or monitor melanosomes (or melanosome-containing compartments) that have been incorporated into keratinocytes. In this study, we performed a comprehensive localization screening for mammalian Rab family small GTPases (Rab1–45) and succeeded in identifying 11 Rabs that were enriched around melanosomes that had been incorporated into keratinocytes. We also established a new assay by using a recently developed melanosome probe (called M-INK) as a means of quantitatively assessing the degradation of proteins on incorporated melanosomes in control and each of a series of Rab-knockdown keratinocytes. The results showed that knockdown or CRISPR/Cas9-mediated knockout of Rab7B (also identified as Rab42) in keratinocytes caused strong inhibition of protein degradation on melanosomes. Our findings indicated that Rab7B/42 is recruited to melanosome-containing compartments and that it promotes protein degradation on melanosomes in keratinocytes.

## Introduction

Melanosomes are specialized organelles that store melanin pigments in mammalian skin melanocytes (Raposo and Marks, 2007). Melanin is synthesized by melanogenic enzymes, e.g., tyrosinase and tyrosinase-related protein 1 (Tyrp1), and deposited within melanosomes. Mature darkly pigmented melanosomes are transported to the periphery of the melanocytes along the cytoskeleton, and they are ultimately transferred to surrounding keratinocytes (Boissy, 2003; Ohbayashi and Fukuda, 2012). After the melanosomes have been incorporated into the keratinocytes, melanin forms supranuclear caps (called “melanin caps”) that protect nuclear DNA against ultraviolet injury (Kobayashi *et al.*, 1998). During the past few decades, a number of proteins involved in the biogenesis and transport of melanosomes in melanocytes have been identified (Ohbayashi and Fukuda, 2012; Bowman *et al.*, 2019), and their molecular mechanisms and functions have gradually been elucidated (Serre *et al.*, 2018). By contrast, the molecular mechanism of melanosome transfer from melanocytes to keratinocytes has remained elusive and is still a matter of controversy, although several transfer models and a related melanosome receptor have been proposed (Seiberg *et al.*, 2000; Ando *et al.*, 2012; Wu *et al.*, 2012; Tarafder *et al.*, 2014; and reviewed in Van Den Bossche *et al.*, 2006; and Tadokoro and Takahashi, 2017). Moreover, subsequent processes such as accumulation and decomposition of the incorporated melanosomes in keratinocytes are poorly understood.

Actually, there have been conflicting reports on the fate or final destination of the melanosomes incorporated into keratinocytes. Some studies have reported that the melanosomes are degraded by autophagy (Murase *et al.*, 2013; Yang *et al.*, 2018; Kim *et al.*, 2019), while others have reported that they reside in nondegradative compartments (Correia *et al.*, 2018; Hurbain *et al.*, 2018). Although several proteins (e.g., LAMP1 [lysosomal-associated membrane protein 1], CD63, EEA1 [early endosomal antigen 1], Rab5B, and LC3) have been observed in melanosome-containing compartments (Murase *et al.*, 2013; Hurbain *et al.*, 2018; Correia *et al.*, 2018), no specific marker protein for such compartments in keratinocytes has been identified, and no appropriate assay to assess the degradation of proteins on melanosomes in keratinocytes has been established.

In this study, we performed a comprehensive localization screening for mouse and human Rab family members (Matsui *et al.*, 2011; Ishida *et al.*, 2012), each of which is thought to localize to a specific membrane compartment(s) (or organelle[s]) and regulates its trafficking (Fukuda, 2008; Pfeffer, 2013; Stenmark, 2009), and succeeded in identifying 11 Rabs that were enriched around melanosomes that had been incorporated into keratinocytes. We then developed a new assay to quantitatively assess the degradation of proteins on such melanosomes in keratinocytes and found that depletion of Rab7B (also identified as Rab42; Itoh *et al.*, 2006; referred to as Rab7B/42 below) in keratinocytes resulted in the strongest inhibition of protein degradation on incorporated melanosomes. Our findings indicated that Rab7B/42 promotes protein degradation on melanosomes in keratinocytes.

## Materials and Methods

### Materials

Anti-Rab7B/42 rabbit polyclonal antibody was produced by using purified GST-tagged mouse Rab7B/42 as described previously (Azouz *et al.*, 2012; Homma *et al.*, 2019). Anti-tyrosinase rabbit polyclonal antibody was prepared as described previously (Beaumont *et al.*, 2011). The following antibodies used in this study were obtained commercially: anti-LAMP1 rat monoclonal antibody (clone: 1D4B) and anti-GM130 mouse monoclonal antibody (clone: 35) (BD Biosciences, San Jose, CA); anti-LBPA (lysobisphosphatidic acid; late endosome marker) mouse monoclonal antibody (clone: 6C4; Echelon Biosciences, Salt Lake City, UT); anti-EEA1 rabbit monoclonal antibody (clone: C45B10; Cell Signaling Technology, Danvers, MA); anti-LC3 rabbit monoclonal antibody (PM036; MBL, Nagoya, Japan); anti-Nogo-A mouse monoclonal antibody (AHP1799; Bio-Rad); anti-Pmel mouse monoclonal antibody (clone: HMB45) (Dako North America, Carpinteria, CA); anti-Tyrp1 mouse monoclonal antibody (clone: Ta99) and anti-Myc mouse monoclonal antibody (clone: 9E10) (Santa Cruz Biotechnology, Santa Cruz, CA); anti-Myc rabbit monoclonal antibody (C3956) and horseradish peroxidase-conjugated anti-FLAG tag (M2) mouse monoclonal antibody (Sigma-Aldrich, St. Louis, MO); anti-RFP (red fluorescent protein) rabbit monoclonal antibody (600-401-379; Rockland, Gilbertsville, PA), anti-β-actin mouse monoclonal antibody (G043; Applied Biological Materials, Richmond, BC, Canada); and Alexa Fluor-labeled secondary antibodies (Thermo Fisher Scientific, Waltham, MA). All other reagents used in this study were analytical grade or the highest grade commercially available.

### Plasmids and small interfering RNAs (siRNAs)

pEGFP-C1 (Takara Bio Inc., Shiga, Japan) vectors carrying mouse Rab1A–43, pEF-FLAG-Rab7B/42, and pMRX-IRES-puro-EGFP-Rab7B/42 were prepared as described previously (Fukuda, 2003; Matsui *et al.*, 2011; Etoh and Fukuda, 2019). The mouse Rab44 and Rab45 cDNAs were amplified from the Marathon-Ready adult brain and testis cDNAs (Takara Bio Inc.) by using specific pairs of oligonucleotides (sequence information available upon request) as described previously (Fukuda, 2003) and then subcloned into the pEGFP-C1 vector. The Rab nomenclature in this study is the nomenclature used in the National Center for Biotechnology Information (NCBI) database, and thus the names of several Rabs, e.g., Rab7B (previously Rab42), Rab42 (previously Rab43), and Rab43 (previously Rab41), differ from the names used in Itoh *et al.* (2006). The M-INK cDNA (Ishida *et al.*, 2017) was subcloned into the pMRX-brs-Myc, pMRX-brs-EGFP, and pMRX-brs-mCherry vectors (Saitoh *et al.*, 2003) (a kind gift from Shoji Yamaoka, Tokyo Medical and Dental University, Tokyo, Japan). Effective siRNAs against mouse Rabs used in this study except *Rab44* siRNA (target sequence: 5'-GAGATCAGCTTGCTTTGA-3') were prepared as described previously (Matsui and Fukuda, 2013). The knockdown efficiency of these siRNAs was checked by co-expressing them with respective EGFP-tagged Rabs in cultured cells (Ishida *et al.*, 2012; Matsui and Fukuda, 2013; and data not shown).

### Cell cultures and transfections

Black-mouse-derived melanocyte cell line melan-a (a generous gift from Dorothy C. Bennett, St. George’s Hospital Medical School, London, UK) (Bennett *et al.*, 1987) and mouse keratinocyte cell line XB2 (purchased from ATCC) were cultured as described previously (Ishida *et al.*, 2017). Plat-E cells (a kind gift from Toshio Kitamura, The University of Tokyo, Japan) and NIH3T3 cells were grown at 37°C in Dulbecco’s modified Eagle medium (044-29765; FUJIFILM Wako Pure Chemical, Osaka, Japan) supplemented with 10% fetal bovine serum, 100 U/ml penicillin G, and 100 μg/ml streptomycin in a 5% CO_2_ incubator. An XB2 cell line stably expressing EGFP (enhanced green fluorescent protein)-Rab7B/42 or NIH3T3 cell lines stably expressing tagged (Myc, EGFP, or mCherry [monomeric Cherry])-M-INK were established by retrovirus infection as described previously (Morita *et al.*, 2000). Plasmids and siRNAs were transfected into XB2 cells by using Lipofectamine 2000 and Lipofectamine RNAiMAX (Thermo Fisher Scientific), respectively, each according to the manufacturer’s protocol.

### CRISPR/Cas9-mediated Rab7B/42 knockout (KO) in XB2 cells

The CRISPR/Cas9-mediated Rab7B/42 KO [single guide RNA target sequence: 5'-TCAGGAGCGGTTCCGCTCAA-3'] in XB2 cells was performed by using pSpCas9 (BB) 2A-Puro vector (ID# 80766; Addgene, Cambridge, MA) and the procedure described previously (Mrozowska and Fukuda, 2016). Clonal lines were isolated and analyzed by immunoblotting with anti-Rab7B/42 antibody to verify the loss of endogenous Rab7B/42 protein expression. *Rab7B/42* gene knockout was confirmed by sequencing genomic PCR products.

### Preparation of melanosomes

Confluent melan-a cells (one 10-cm dish) were scraped off the culture dish, homogenized in a homogenization buffer (0.25 M sucrose, 10 mM HEPES-KOH, pH 7.2, 1 mM 2-mercaptoethanol, and 1 mM EDTA) by passing them through a 27-gauge needle 5 times, and then centrifuged at 500×*g* for 1 minute. After collecting the supernatants and re-centrifuging at 10,000×*g* for 3 minutes, the pellets were washed twice with the homogenization buffer and centrifuged again at 10,000×*g* for 3 minutes. The pellets were then re-suspended in the homogenization buffer, frozen with liquid nitrogen, and stored at –80°C before use. The absorbance of the melanosome-rich solution was measured with a BioPhotometer D30 (Eppendorf, Hamburg, Germany) at 340 nm, and the melanosome concentration was calculated according to a calibration curve developed in-house (absorbance=2.9876×concentration [μg/μl]–0.2854).

### Preparation of M-INK-containing lysates

Confluent M-INK-expressing NIH3T3 cells (one 10-cm dish) were scraped off the culture dish, homogenized in 1 ml of phosphate-buffered saline (PBS) by passing through a 27-gauge needle 3 times, and centrifuged at 17,000×*g* for 5 minutes. The supernatants were collected and used as M-INK-containing lysates (simply referred to as M-INK lysates below). The M-INK lysates were frozen with liquid nitrogen and stored at –80°C before use.

### Uptake of melanosomes by keratinocytes

XB2 cells were cultured on 24-well plates containing a 15-mm diameter coverslip, and 50 μg of purified melanosomes were added per 500 μl of culture medium. After incubation for 48 hours, the cells were fixed with 4% paraformaldehyde. The coverslips were incubated with a 100 μl volume of M-INK lysates (containing complete EDTA-free protease inhibitor cocktail [Roche Applied Science, Penzberg, Germany] and 0.05% saponin), then with primary antibodies and lastly with appropriate Alexa Fluor-conjugated secondary antibodies. The samples were mounted using ProLong Diamond Antifade Mountant (Thermo Fisher Scientific) and examined for fluorescence with a confocal fluorescence microscope (FV1000D, Olympus, Tokyo, Japan).

### M-INK degradation assay

Purified melanosomes were mixed with M-INK lysate (melanosome:M-INK lysate=1 μg:1 μl), incubated for 10 minutes, and then centrifuged at 10,000×*g* for 1 minute. The pellets were re-suspended with the culture medium and used immediately as M-INK-labeled melanosomes. After culturing XB2 cells with M-INK-labeled melanosomes, the cells were washed twice with PBS, collected by trypsinization, and centrifuged at 500×*g* for 1 minute. The cell pellets were lysed in an SDS sample buffer (62.5 mM Tris-HCl pH 6.8, 2% 2-mercaptoethanol, 10% glycerol, and 0.02% Bromophenol Blue) and boiled. The lysates were subjected to SDS-PAGE and transferred to a polyvinylidene difluoride membrane (Merck Millipore, Burlington, MA) by electroblotting. The blots were blocked with 5% skim milk in PBS containing 0.1% Tween-20 (FUJIFILM Wako Pure Chemical), incubated at room temperature with primary antibodies for 1 hour and then with appropriate horseradish peroxidase-conjugated secondary antibodies for 1 hour. Immunoreactive bands were detected by using the ChemiDoc Touch imaging system (Bio-Rad, Hercules, CA) and quantified with ImageJ software (version 1.52b; National Institutes of Health).

### Assay for degradation of melanosomal proteins in keratinocytes

XB2 cells were cultured on a 10-cm dish (or 6-cm dish for Fig. S4) with or without 1 mg of purified melanosomes. After incubation for 36 hours, the cells were washed twice with PBS, collected by trypsinization, and centrifuged at 500×*g* for 1 minute. The cell pellets were suspended in the homogenization buffer (containing complete EDTA-free protease inhibitor cocktail), homogenized by passing them through a 27-gauge needle 30 times, and then centrifuged at 3000×*g* for 5 minutes. The pellets were lysed in the SDS sample buffer, vortexed with 0.5 mm glass beads for 30 minutes at 4°C, and boiled for 15 minutes. The lysates obtained were analyzed by immunoblotting with anti-tyrosinase antibody and anti-Pmel antibody.

### Live-cell imaging

Live-cell fluorescence imaging was performed by using a FV1000D confocal fluorescence microscope with a 100×oil/1.4 NA Plan Apochromatic objective lens and Fluoview software. XB2 cells stably expressing EGFP-Rab7B/42 were placed on a 35-mm glass bottom dish (MatTek, Ashland, MA) and incubated for 2 hours with mCherry-M-INK-labeled melanosomes before imaging. During live-cell imaging, the dish was mounted in a chamber (INUB-ONI-F2; Tokai Hit Co., Ltd., Shizuoka, Japan) to maintain incubation conditions at 37°C and 5% CO_2_. Images were acquired at intervals of 10 minutes and analyzed with ImageJ software.

### Melanin assay

Melanin content was assayed as described previously (Tamura *et al.*, 2009). In brief, XB2 cells were cultured for 48 hours with melanosomes, washed two times with PBS, then cultured for 2 weeks in the absence of melanosomes. Their melanin content normalized to protein content was measured as optical density at 490 nm.

## Results

### Melanosomes incorporated into keratinocytes are surrounded by LAMP1-positive and LysoTracker-negative structures

**Fig. 1:**
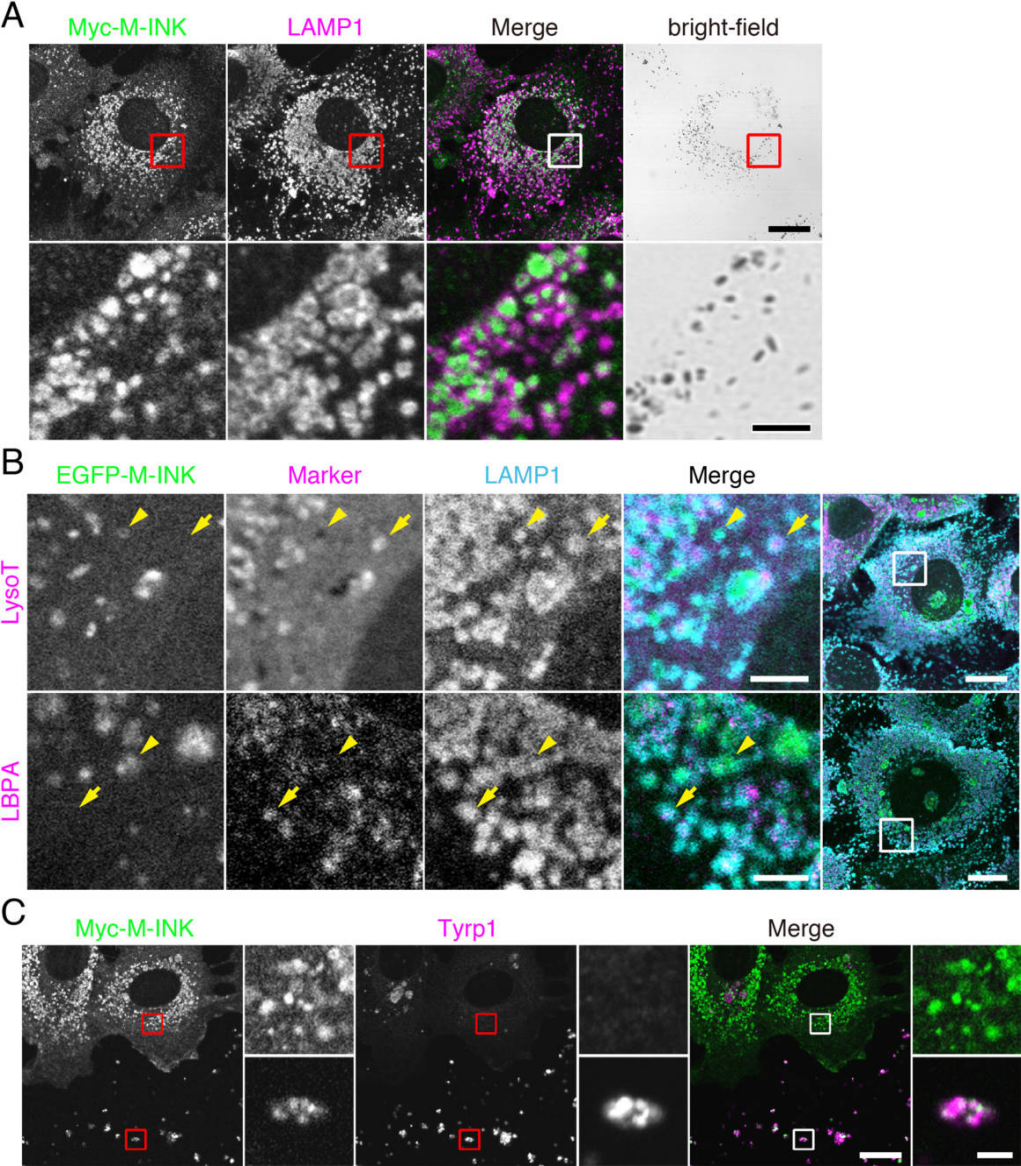
Melanosomes incorporated by keratinocytes are surrounded by LAMP1-positive structures. XB2 cells were cultured with melanosomes for 48 hours. (A) The cells were stained for Myc-M-INK (green) and LAMP1 (magenta). (B) LysoTracker Red (LysoT) was added 15 minutes before fixation. The cells were stained for EGFP-M-INK (green), LBPA (magenta), and LAMP1 (cyan). The arrowheads point to structures positive for both M-INK and LAMP1. The arrows point to M-INK-negative, LAMP1-positive structures. (C) The cells were stained for Myc-M-INK (green) and Tyrp1 (magenta). Upper insets, Tyrp1-negative melanosomes in keratinocytes; and lower insets, Tyrp1-positive extracellular melanosomes. Scale bars=15 μm (3 μm in magnified views of A and B, and 2 μm in magnified views of C).

To identify a membrane compartment containing melanosomes in mouse XB2 keratinocytes, we co-stained XB2 cells that had been cultured with isolated melanosomes for 48 hours for various organelle markers and melanosomes. We stained for melanosomes by using the recently developed M-INK (Melanocore-INteracting Kif1c-tail) probe, which enables visualization of melanosomes in a fluorescent field (Ishida *et al.*, 2017). Fluorescence visualization with M-INK is more accurate in the z-axis direction than bright-field observation, and M-INK makes it possible to correctly determine the intracellular position of melanosomes that have been incorporated into keratinocytes. Conventional markers for melanosomes (e.g., tyrosinase and Tyrp1) in melanocytes cannot be used, because they are not stained in keratinocytes that have incorporated melanosomes (Fig. 1C; Ishida *et al.*, 2017). The results of the co-staining analysis showed that many of the melanosomes were surrounded by LAMP1-positive structures (Fig. 1A), but they were not well colocalized with other organelle markers, including EEA1 (early endosome marker), LBPA, LysoTracker Red (LysoT; lysosome marker), LC3 (autophagosome marker) and Nogo-A (ER marker) (Supplemental Fig. S1). Because LAMP1 is known to localize both in lysosomes and late endosomes (Humphries *et al.*, 2011), we also co-stained XB2 cells for LAMP1, LBPA (or LysoTracker Red), and melanosomes. Intriguingly, no M-INK-positive, LAMP1-positive structures were co-localized with LysoTracker Red or LBPA (Fig. 1B, arrowheads). Such LAMP1-positive, LysoTracker-negative localization of melanosomes in keratinocytes has also recently been described in another study (Correia *et al.*, 2018; Hurbain *et al.*, 2018). These findings indicated that the melanosomes in keratinocytes are present in a subset of LAMP1-positive structures that are different from both highly acidic, degradative lysosomes (i.e., LysoTracker-positive lysosomes) and conventional late endosomes (i.e., LBPA-positive late endosomes).

**Fig. 2:**
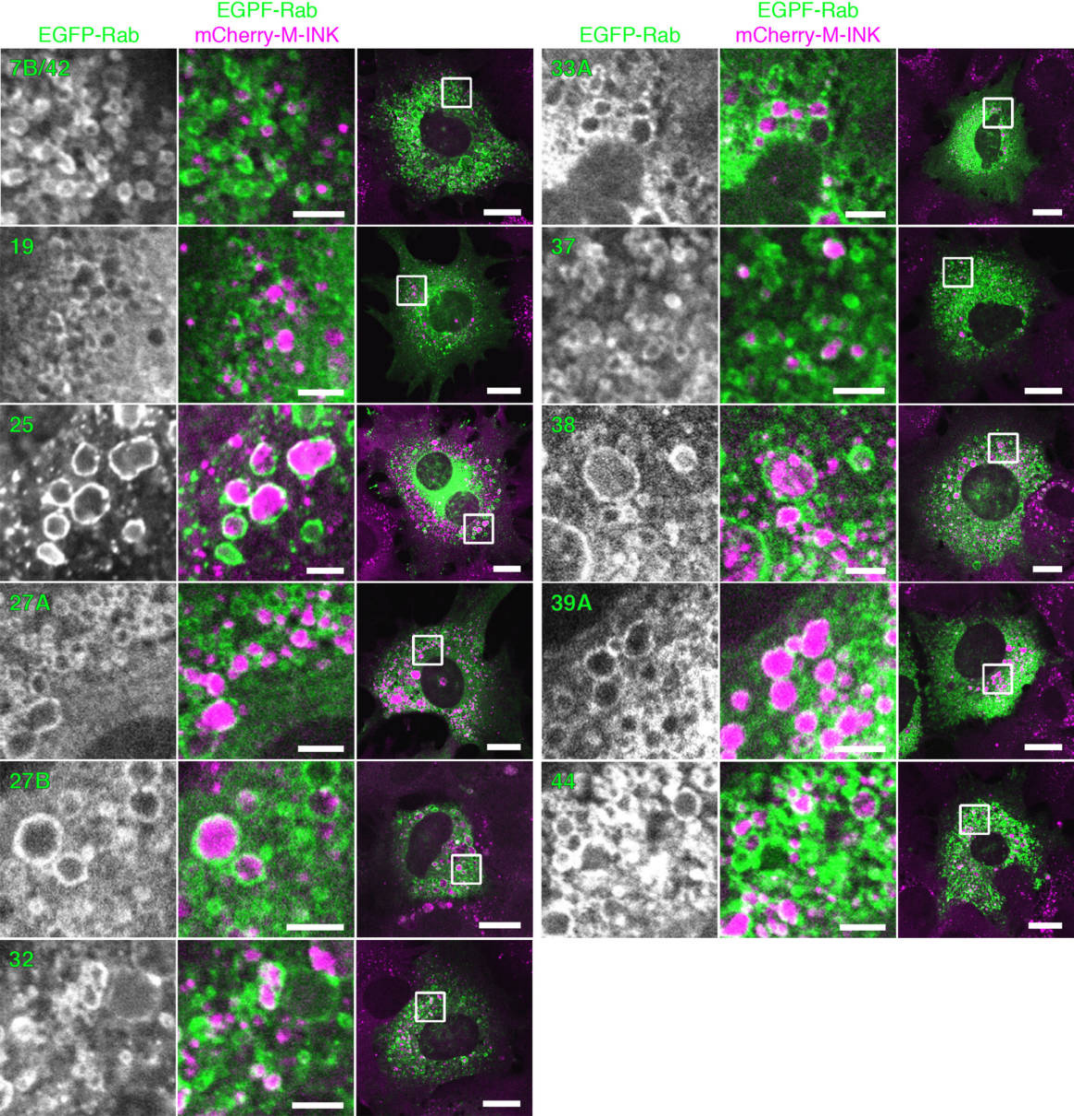
Rabs that are specifically localized around melanosomes incorporated into keratinocytes. XB2 cells expressing EGFP-Rabs (Rab1A–45; green) were stained for mCherry-M-INK (magenta). Only 11 of the Rabs were enriched around the incorporated melanosomes, and none of the other Rabs were localized around the incorporated melanosomes (see Supplemental Fig. S2). Scale bars=15 μm (3 μm in magnified views).

To further characterize the LAMP1-positive structures around the incorporated melanosomes, we focused on the Rab family small GTPases, because approximately 60 different isoforms are present in mammals and each member is thought to localize to a specific membrane compartment(s) or organelle(s) (Fukuda, 2008; Pfeffer, 2013; Stenmark, 2009). We expressed each of 62 different EGFP-tagged Rabs (Rab1A–45) (Matsui *et al.*, 2011; Ishida *et al.*, 2012) in XB2 cells, and the incorporated melanosomes were visualized with M-INK. The results of a comprehensive Rab localization screening revealed that 11 Rabs, i.e., Rab7B/42, 19, 25, 27A, 27B, 32, 33A, 37, 38, 39A, and 44, were enriched around the incorporated melanosomes (Fig. 2) and that none of the other Rabs were localized around them (Supplemental Fig. S2). Intriguingly, neither early endosomal Rab5B, which has previously been shown to regulate melanosome uptake in keratinocytes (Correia *et al.*, 2018), late endosomal/lysosomal Rab7A, which is involved in the degradation of endocytosed receptors (Guerra and Bucci, 2016), nor recycling endosomal Rab11B, which is required for melanosome transfer from melanocytes to keratinocytes (Tarafder *et al.*, 2014), was among the 11 candidate Rabs identified by screening. We therefore speculated that melanosomes that have been incorporated into keratinocytes are present in an as yet unidentified Rab-positive, LAMP1-positive compartment.

### An incorporated-melanosome-containing structure is a degradative compartment: establishment of an assay for M-INK degradation in keratinocytes

**Fig. 3:**
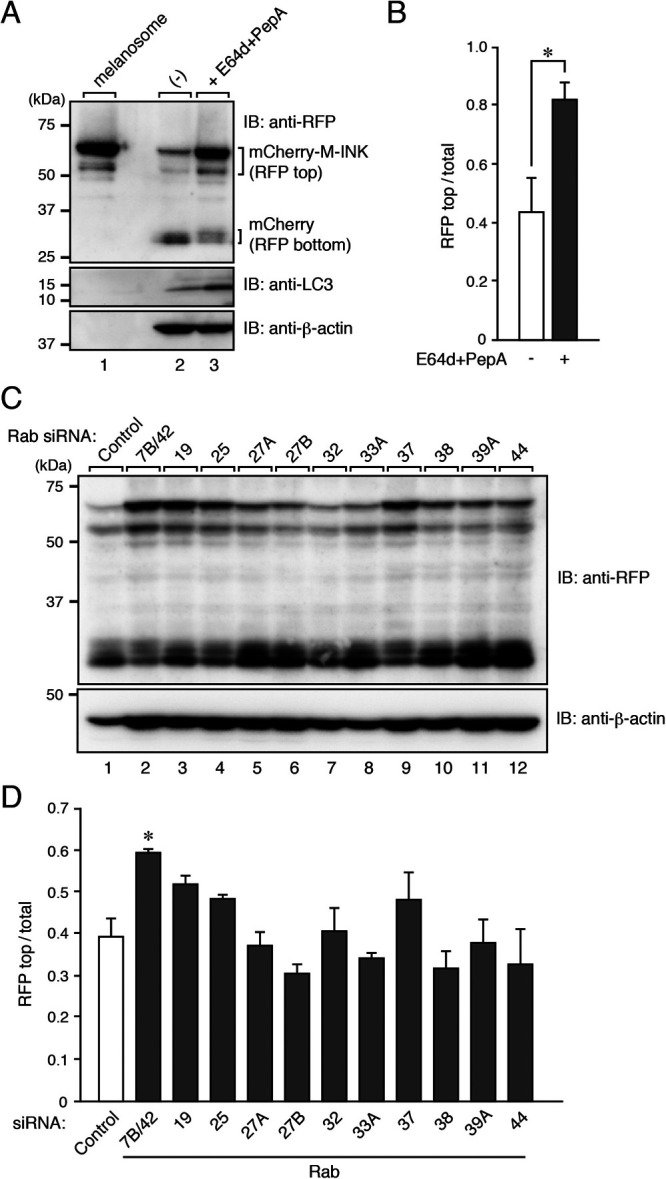
Degradation of M-INK in Rab-KD XB2 cells. (A) XB2 cells were cultured with mCherry-M-INK-labeled melanosomes for 24 hours in the presence or absence of E64d and pepstatin A and assessed for degradation of M-INK by immunoblotting. (B) Quantification of RFP-positive bands shown in the top panel in A. The total amount of RFP/mCherry was calculated by adding the top and bottom RFP-positive bands (mean+s.e.m.; n=3). *, *p*<0.05 (Student’s unpaired *t* test). (C) XB2 cells were treated with control or *Rab* siRNAs and then cultured with mCherry-M-INK-labeled melanosomes for 24 hours. The cell lysates were analyzed by immunoblotting. (D) Quantification of RFP-positive bands shown in the top panel in C (mean+s.e.m.; n=3). *, *p*<0.05 compared with the control (Dunnett’s test).

Although no incorporated melanosomes were detected in LysoTracker-positive lysosomes (Fig. 1B), Tyrp1 signals largely disappeared when melanosomes were incorporated into XB2 cells (Fig. 1C, upper insets), suggesting that the incorporated-melanosome-containing structures in keratinocytes still had degradative activity. Because melanogenic enzymes such as tyrosinase and Tyrp1 are rapidly degraded in keratinocytes (Ishida *et al.*, 2017), it is extremely difficult to monitor their degradation over a long period. To overcome this problem, we developed an M-INK degradation assay (see Materials and Methods for details) in order to be able to quantitatively assess protein degradation on melanosomes in keratinocytes. In brief, we pre-labeled melanosomes with mCherry-M-INK, incorporated them into keratinocytes, and then detected the mCherry-M-INK in the keratinocytes by immunoblotting with anti-RFP antibody. Although the amount of full-length incorporated mCherry-M-INK was much lower (Fig. 3A; compare lanes 1 and 2; ~60 kDa band), the mCherry band itself seemed to have been protected even after incubating the melanosomes in XB2 cells for 24 hours (Fig. 3A; ~30 kDa band), presumably because mCherry is less susceptible to degradation than M-INK is. It should be noted that the degradation of M-INK was almost completely inhibited by treatment with the protease inhibitors E64d and pepstatin A (Fig. 3A, lane 3). We therefore decided to use the ratio of mCherry-M-INK to mCherry as an index of melanosomal protein degradation, and we succeeded in quantitatively comparing the degradation of mCherry-M-INK in the presence and absence of protease inhibitors (Fig. 3B).

We used our assay to investigate the candidate Rabs that surrounded the incorporated melanosomes (Fig. 2) for involvement in protein degradation on melanosomes. We knocked down each candidate Rab in XB2 cells with specific siRNAs (Matsui and Fukuda, 2013) and evaluated the degradation ability of each Rab-knockdown (KD) cells. The results showed that knockdown of Rab7B/42 most strongly and significantly inhibited M-INK degradation (Fig. 3C and D). Rab7B/42 has previously been reported to be localized at different organelles in other cell types, e.g., at late endosomes or the *trans*-Golgi network (TGN) in HeLa cells (Progida *et al.*, 2010; Progida *et al.*, 2012) and late endosomes or lysosomes in immune cells (Yang *et al.*, 2004; Wang *et al.*, 2007; Yao *et al.*, 2009), and to be implicated in the trafficking and degradation of certain immune receptors (Wang *et al.*, 2007; Yao *et al.*, 2009), cathepsin-D maturation (Progida *et al.*, 2010), and autophagy (Kjos *et al.*, 2017). Based on these previous observations together with our own finding that Rab7B/42 is strongly colocalized with LAMP1 but weakly colocalized with LysoTracker Red or LBPA in XB2 cells (Supplemental Fig. S3), we selected Rab7B/42 as the prime candidate for subsequent analysis as the Rab that regulates protein degradation on melanosomes.

### Rab7B/42 promotes protein degradation on melanosomes in keratinocytes

**Fig. 4:**
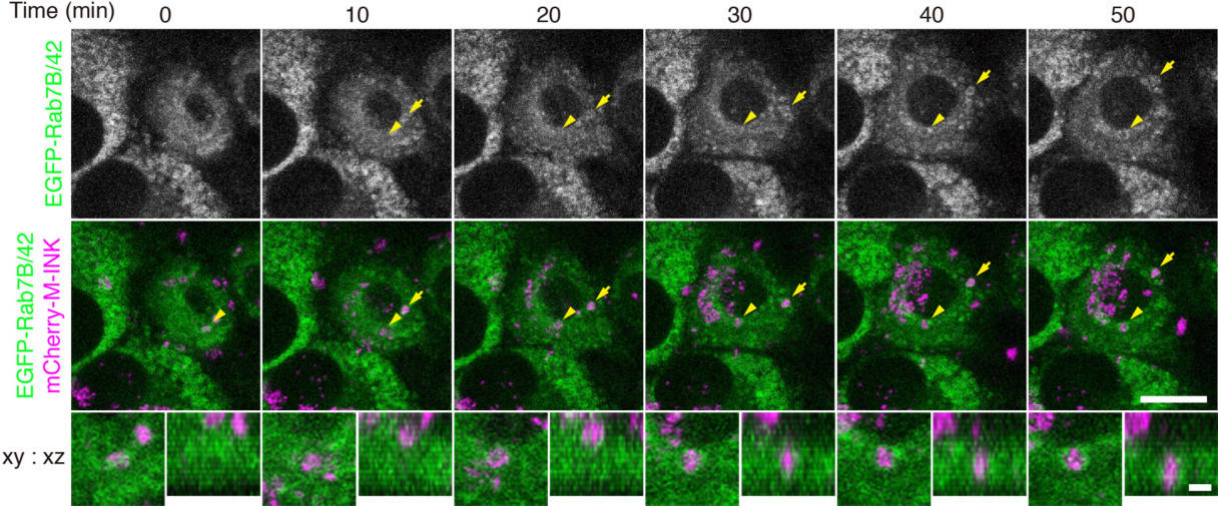
EGFP-Rab7B/42 accumulates around melanosomes incorporated into keratinocytes. Live imaging of EGFP-Rab7B/42 stably expressing in XB2 cells after adding mCherry-M-INK-labeled melanosomes. The images were stacked by z-projection. The arrowheads and arrows point to EGFP-Rab7B/42 accumulations around incorporated melanosomes (see also Supplemental Movies 1 and 2). Magnified views are stacked images of the incorporated melanosomes pointed to the arrowheads. Scale bars=15 μm (2 μm in magnified views).

To investigate the timing and position of Rab7B/42 recruitment to melanosomes in keratinocytes in greater detail, we performed live-cell imaging of XB2 cells stably expressing EGFP-Rab7B/42 in the presence of mCherry-M-INK-labeled melanosomes. Examination of serial section images revealed that EGFP-Rab7B/42 gradually accumulated around the melanosomes (Fig. 4, arrows and arrowheads; and Supplemental Movies 1 and 2).

**Fig. 5:**
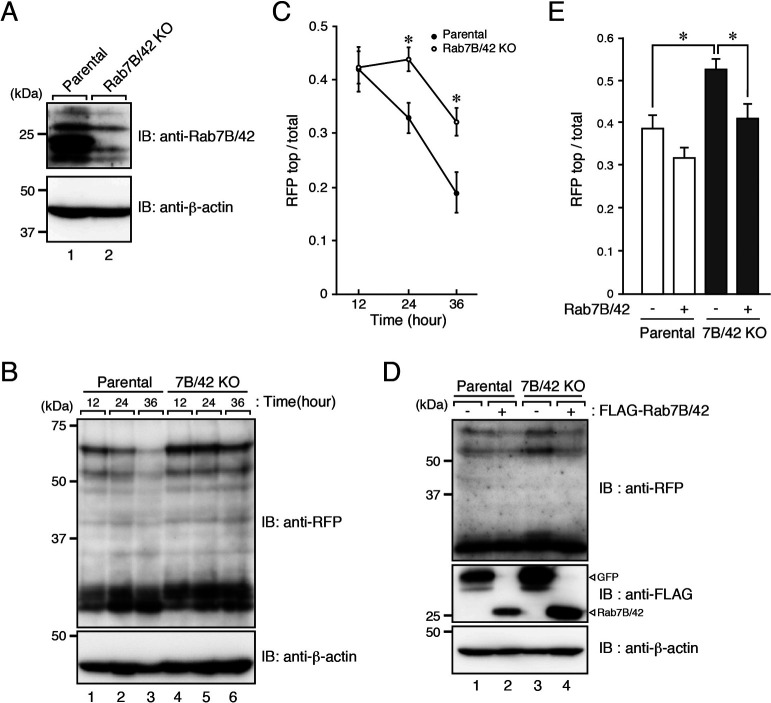
Impaired M-INK degradation in Rab7B/42-KO XB2 cells. (A) Expression of Rab7B/42 in parental and Rab7B/42-KO XB2 cells. (B) Parental and Rab7B/42-KO cells were incubated for 12 hours with mCherry-M-INK-labeled melanosomes and then cultured for the times indicated in the absence of melanosomes. The cell lysates were analyzed by immunoblotting. (C) Quantification of RFP-positive bands shown in the top panel in B (mean+s.e.m.; n=3). *, *p*<0.05 (Student’s unpaired *t* test). (D) Parental and Rab7B/42-KO cells expressing FLAG-Rab7B/42 (or FLAG-GFP) were cultured with mCherry-M-INK-labeled melanosomes for 24 hours, and immunoblotting was performed. (E) Quantification of RFP-positive bands shown in the top panel in D (mean+s.e.m.; n=10). *, *p*<0.05 (Tukey’s test).

We then generated a Rab7B/42-KO cell line by using the CRISPR/Cas9 system, and we succeeded in confirming the loss of its protein by immunoblotting with specific antibody (Fig. 5A). To evaluate the degradation ability of Rab7B/42-KO cells, we incubated the cells with mCherry-M-INK-labeled melanosomes and investigated the degradation of mCherry-M-INK by immunoblotting with anti-RFP antibody as described in Fig. 3A. Consistent with the results of Rab7B/42 KD shown in Fig. 3D, Rab7B/42 KO caused a significant and clear delay of protein degradation on the melanosomes in the keratinocytes (Fig. 5B and C). Moreover, the inhibition of M-INK degradation in the absence of Rab7B/42 was clearly rescued by re-expression of Rab7B/42 in Rab7B/42-KO cells (Fig. 5D and E).

**Fig. 6:**
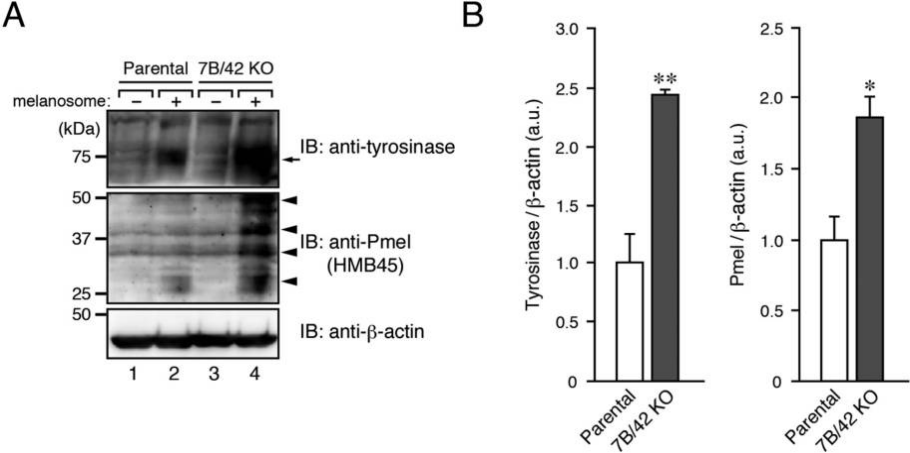
Rab7B/42-KO in keratinocytes inhibits melanosomal protein degradation. (A) Parental and Rab7B/42-KO cells were incubated for 36 hours with or without melanosomes. The lysates of the melanosome-containing compartment or the total cell lysates (for β-actin) were analyzed by immunoblotting with the antibodies indicated. (B) Quantification of tyrosinase (arrow) and Pmel bands (arrowheads) shown in the top and middle panels, respectively, in A (mean+s.e.m.; n=3). *, *p*<0.05; **, *p*<0.01 (Student’s unpaired *t* test).

Finally, we evaluated the degradation of endogenous melanosomal proteins, i.e., tyrosinase and Pmel (also called Pmel17/gp100), in parental and Rab7B/42-KO cells by incubating the cells with or without melanosomes. The results showed that Rab7B/42 KO significantly inhibited the degradation of the two melanosomal proteins (Fig. 6), consistent with the impaired M-INK degradation shown in Fig. 5. Such inhibitory effects are unlikely to be caused by clonal variations of XB2 cells, because inhibition of tyrosinase and Pmel degradation was also observed in another independent Rab7B/42 KO#2 clone and because it was clearly rescued by re-expression of Rab7B/42 (Supplemental Fig. S4). These results indicated that Rab7B/42 promotes protein degradation on melanosomes in keratinocytes.

## Discussion

In the present study, we used a collection of EGFP-tagged Rabs together with an M-INK probe and succeeded in demonstrating that melanosomes that have been incorporated into mouse XB2 keratinocytes are surrounded by structures that are LAMP1- and Rab7B/42-positive, but LysoTracker-negative (Fig. 1, Fig. 2, and Fig. 4). We also found that depletion of Rab7B/42 in XB2 cells caused strong inhibition of protein degradation on incorporated melanosomes (Fig. 3, Fig. 5, and Fig. 6), but that M-INK degradation in Rab7B/42-KO cells was not completely blocked (Fig. 5C, 36 hours). This residual degradation activity may be mediated by other Rab isoforms that are also present around melanosomes. Actually, our screening procedure identified 10 additional Rabs (i.e., Rab19, 25, 27A, 27B, 32, 33A, 37, 38, 39A, and 44), and some of them have been shown to be involved in related processes in other cell types; e.g., Rab27A and Rab32 have been shown to be recruited around internalized pathogens and involved in subsequent clearance processes (Yokoyama *et al.*, 2011; Spanò *et al.*, 2016). Further research will be necessary to determine the function of these Rabs in the uptake, transport, and/or decomposition of melanosomes in keratinocytes. Intriguingly, two well-known Rabs, Rab5B, which regulates melanosome uptake in keratinocytes (Correia *et al.*, 2018), and Rab7A, which generally regulates the endocytic pathway (Guerra and Bucci, 2016), were not identified by our screening procedure (Supplemental Fig. S2). The absence of Rab5B in the candidate list is probably attributable to having imaged keratinocytes 48 hours after the addition of melanosomes, which is much later than the time when melanosome uptake occurs. Although Rab7B is the closest isoform of Rab7A, Rab7A itself is not recruited around incorporated melanosomes and is unlikely to play a major role in protein degradation on melanosomes, suggesting the existence of functional diversity between two Rab7 isoforms in keratinocytes.

How does Rab7B/42 promote protein degradation on melanosomes in keratinocytes? Because Rab7B/42 has been shown to be involved in cathepsin-D maturation (Progida *et al.*, 2010) and Rab7B/42 is well colocalized with LAMP1 in keratinocytes (Supplemental Fig. S3), Rab7B/42 is likely to regulate the trafficking of certain E64d- and pepstatin A-sensitive proteases to LAMP1-positive, LysoTracker-negative compartments. Identification of proteases that degrade proteins on melanosomes at relatively higher pH’s, at which LysoTracker is negative, is an important task that needs to be addressed in a future study. Although the Rab7B/42-positive melanosome-containing compartments identified in our study have the ability to degrade proteins on melanosomes (e.g., tyrosinase and Pmel; Fig. 6 and Supplemental Fig. S4), no melanin itself had been degraded in either parental or Rab7B/42-KO cells even after two weeks (Supplemental Fig. S5), suggesting that melanin degradation occurs in a compartment(s) other than Rab7B/42- and LAMP1-positive compartments. One possible degradation mechanism is autophagy, but LC3 was not well colocalized with the incorporated melanosomes in our study (Supplemental Fig. S1). However, since the presence of LC3 has been reported in the upper layers of the epidermis (Akinduro *et al.*, 2016; Hurbain *et al.*, 2018), keratinocyte differentiation may regulate autophagic activity and promote melanin degradation.

In conclusion, we have demonstrated that incorporated melanosomes are present in Rab7B/42- and LAMP1-positive compartments and that Rab7B/42 can promote protein degradation on melanosomes without altering the melanin content of keratinocytes. Our findings provide new insights into the degradation processes of melanosomes in keratinocytes, and the M-INK degradation assay developed in this study will be useful in analyzing the precise molecular mechanism of protein degradation on melanosomes in the future.

## Abbreviations

EEA1: early endosomal antigen 1
EGFP: enhanced green fluorescent protein
KD: knockdown
KO: knockout
LAMP1: lysosomal-associated membrane protein 1
LBPA: lysobisphosphatidic acid
LysoT: LysoTracker Red
mCherry: monomeric Cherry
M-INK: Melanocore-INteracting Kif1c-tail
PBS: phosphate-buffered saline
RFP: red fluorescent protein
siRNA: small interfering RNA
Tyrp1: tyrosinase-related protein 1

## Supplementary Material

Supplemental Fig. S1 Melanosomes incorporated into keratinocytes are not well colocalized with organelle markers except LAMP1 (related to Fig. 1). XB2 cells were cultured with melanosomes for 48 hours. The cells were stained for Myc-M-INK (green) and the organelle markers indicated (magenta). LysoTracker Red (LysoT) was added 15 minutes before fixation. Scale bars=15 μm (3 μm in magnified views).

Supplemental Fig. S2 Screening for EGFP-Rabs that are specifically localized around melanosomes incorporated into XB2 cells (related to Fig. 2). XB2 cells expressing EGFP-Rabs (Rab1A–45; green) were stained for mCherry-M-INK (magenta). Rabs not efficiently recruited to incorporated melanosomes are shown. Scale bars=15 μm (3 μm in magnified views).

Supplemental Fig. S3 Subcellular localization of Rab7B/42 in keratinocytes (related to Fig. 4). EGFP-Rab7B/42-expressing XB2 cells were stained for EGFP (green) and several organelle markers, including LAMP1, LysoTracker Red (LysoT), LBPA, and GM130 (magenta). Scale bars=15 μm (3 μm in magnified views).

Supplemental Fig. S4 Inhibition of melanosomal protein degradation in another independent Rab7B/42-KO#2 clone. (A) Parental, Rab7B/42-KO#2, and Rab7B/42-KO#2 cells stably expressing EGFP-Rab7B/42 were incubated for 36 hours with melanosomes. The lysates of the melanosome-containing compartment or the total cell lysates (for Rab7B/42 and β-actin) were analyzed by immunoblotting with the antibodies indicated. Two higher bands of Pmel (~37 kDa and ~50 kDa; see Fig. 6A) were not evident, presumably because the amount of cell lysates loaded in Fig. S4A was smaller than that in Fig. 6A. (B) Quantification of tyrosinase (arrow) and Pmel bands (arrowheads) shown in the top and second panels, respectively, in A (mean+s.e.m.; n=3). *, *p*<0.05; **, *p*<0.01 (Tukey’s test).

Supplemental Fig. S5 Melanin content of keratinocytes after melanosome uptake (related to Fig. 5). Parental and Rab7B/42-KO cells were incubated for 48 hours with melanosomes and then cultured for two weeks in the absence of melanosomes. Their melanin content was measured as optical density. The bars represent the means+s.e.m. of the data obtained in three independent experiments.

Supplemental Movie 1 EGFP-Rab7B/42 accumulates around melanosomes in keratinocytes (xy view of Fig. 4). XB2 cells stably expressing EGFP-Rab7B/42 were incubated for 2 hours with mCherry-M-INK-labeled melanosomes, and then images were acquired at intervals of 10 minutes.

Supplemental Movie 2 EGFP-Rab7B/42 accumulates around melanosomes in keratinocytes (magnified xyz view of Fig. 4). XB2 cells stably expressing EGFP-Rab7B/42 were incubated for 2 hours with mCherry-M-INK-labeled melanosomes, and then images were acquired at intervals of 10 minutes.
