# Supplementary figures and images for: Rab7B/42 Is Functionally Involved in Protein Degradation on Melanosomes in Keratinocytes
Source: Cell Struct Funct. 2020 Feb 7;45(1):45–55. doi: 10.1247/csf.19039 (PMC10739166; doi:10.1247/csf.19039)

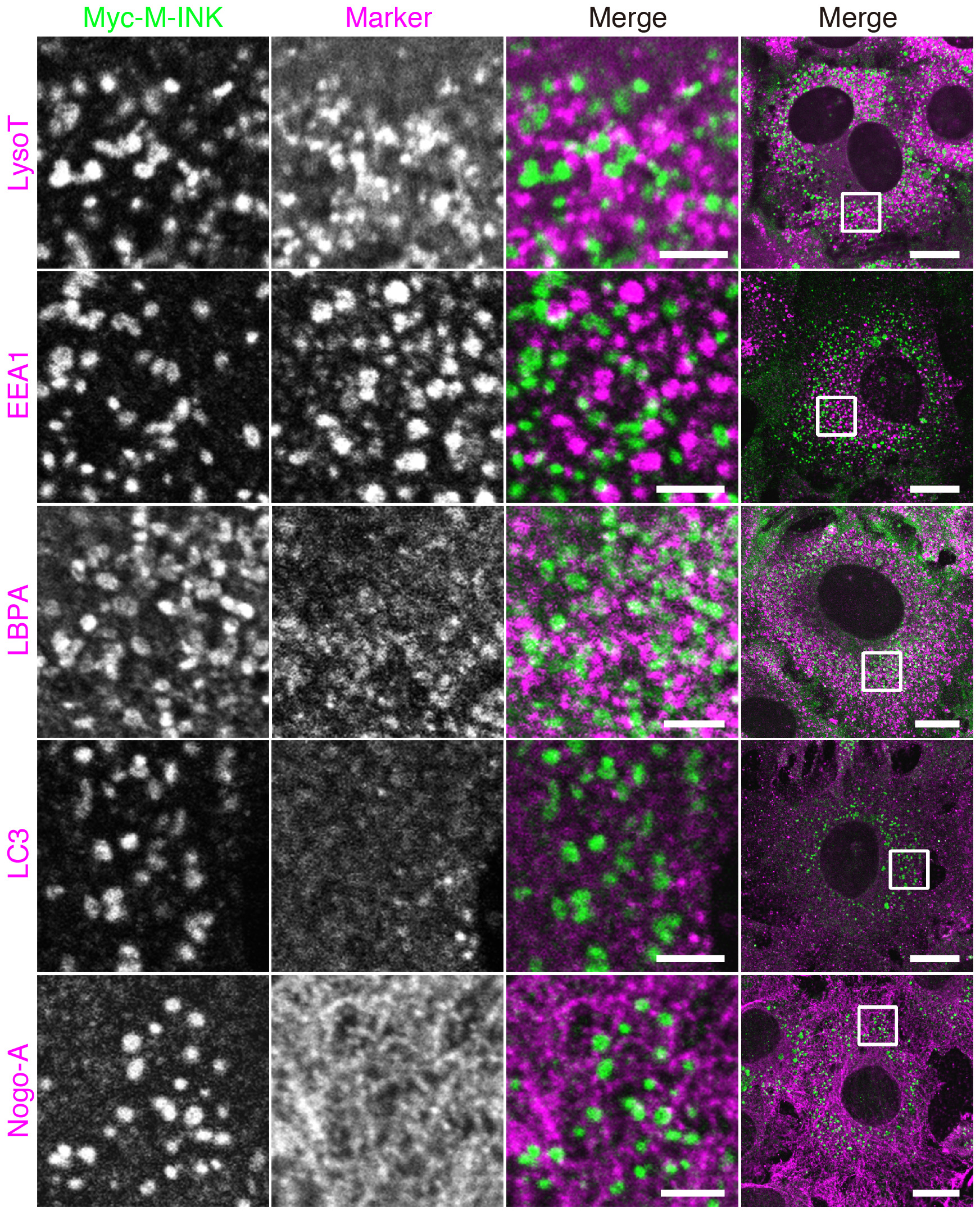

Supplement: Supplementary file 1 — Supplemental Fig. S1 Melanosomes incorporated into keratinocytes are not well colocalized with organelle markers except LAMP1 (related to Fig. 1). XB2 cells were cultured with melanosomes for 48 hours. The cells were stained for Myc-M-INK (green) and the organelle markers indicated (magenta). LysoTracker Red (LysoT) was added 15 minutes before fixation. Scale bars=15 μm (3 μm in magnified views). [file csf_45_19039_1.jpg]

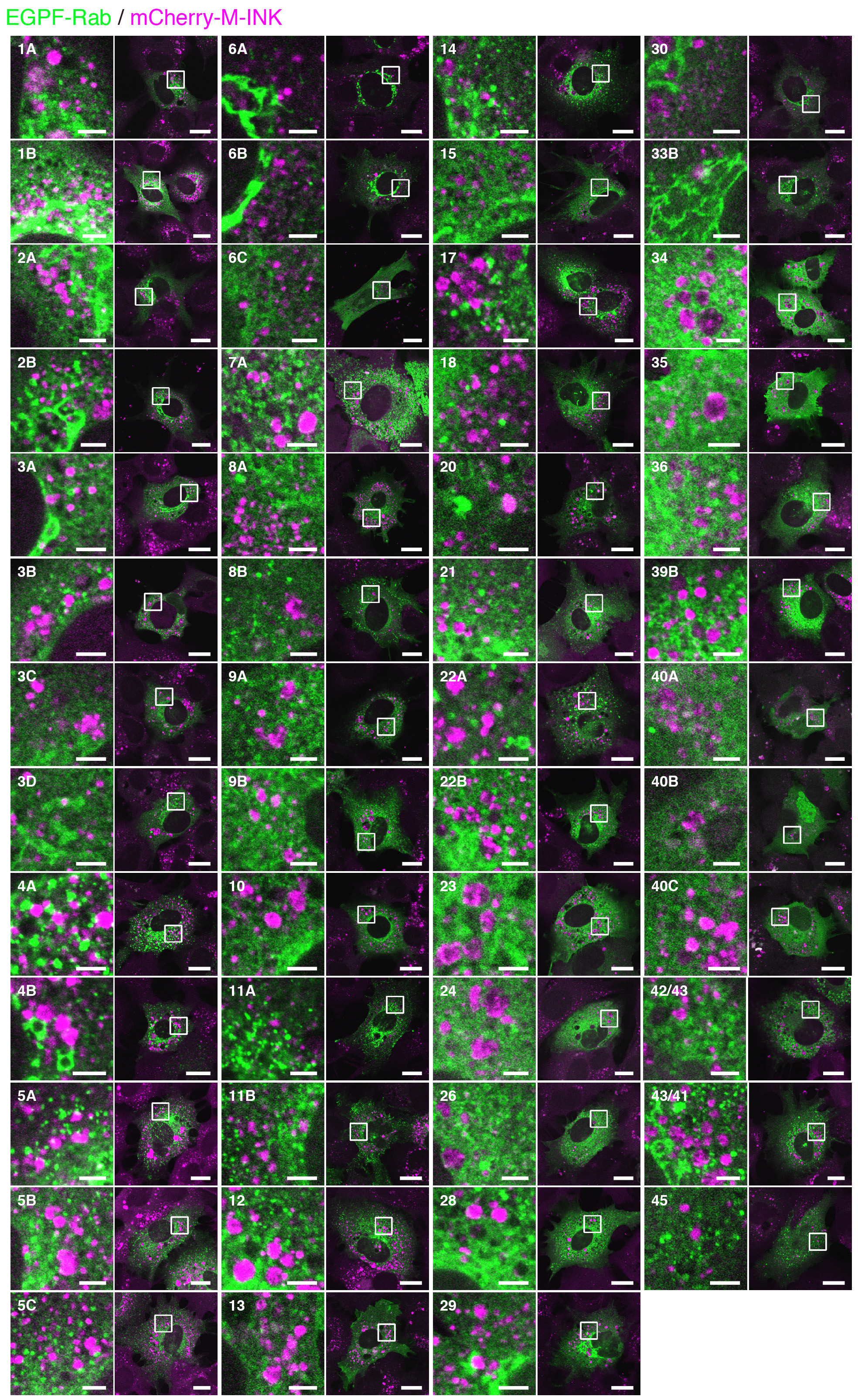

Supplement: Supplementary file 2 — Supplemental Fig. S2 Screening for EGFP-Rabs that are specifically localized around melanosomes incorporated into XB2 cells (related to Fig. 2). XB2 cells expressing EGFP-Rabs (Rab1A–45; green) were stained for mCherry-M-INK (magenta). Rabs not efficiently recruited to incorporated melanosomes are shown. Scale bars=15 μm (3 μm in magnified views). [file csf_45_19039_2.jpg]

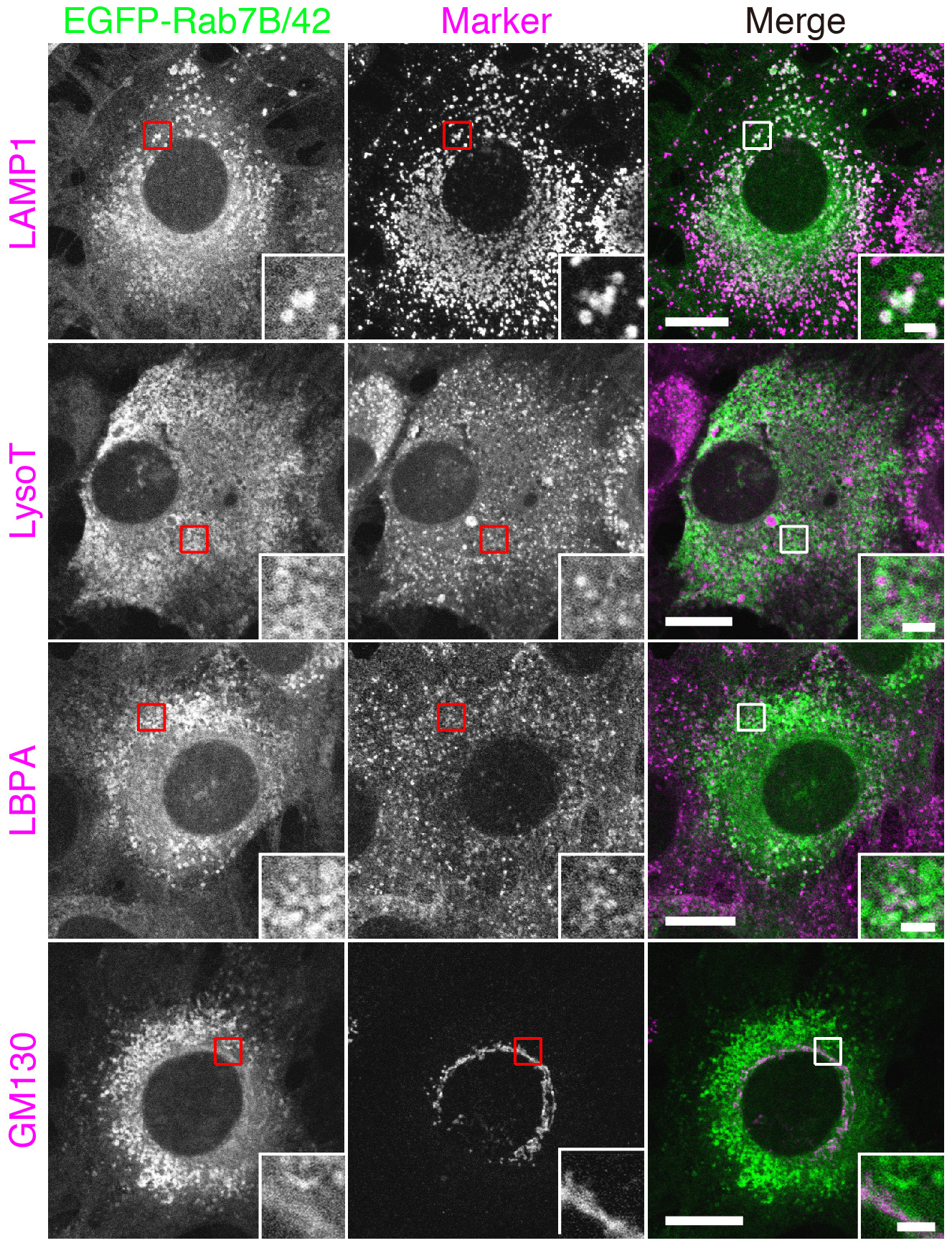

Supplement: Supplementary file 3 — Supplemental Fig. S3 Subcellular localization of Rab7B/42 in keratinocytes (related to Fig. 4). EGFP-Rab7B/42-expressing XB2 cells were stained for EGFP (green) and several organelle markers, including LAMP1, LysoTracker Red (LysoT), LBPA, and GM130 (magenta). Scale bars=15 μm (3 μm in magnified views). [file csf_45_19039_3.jpg]

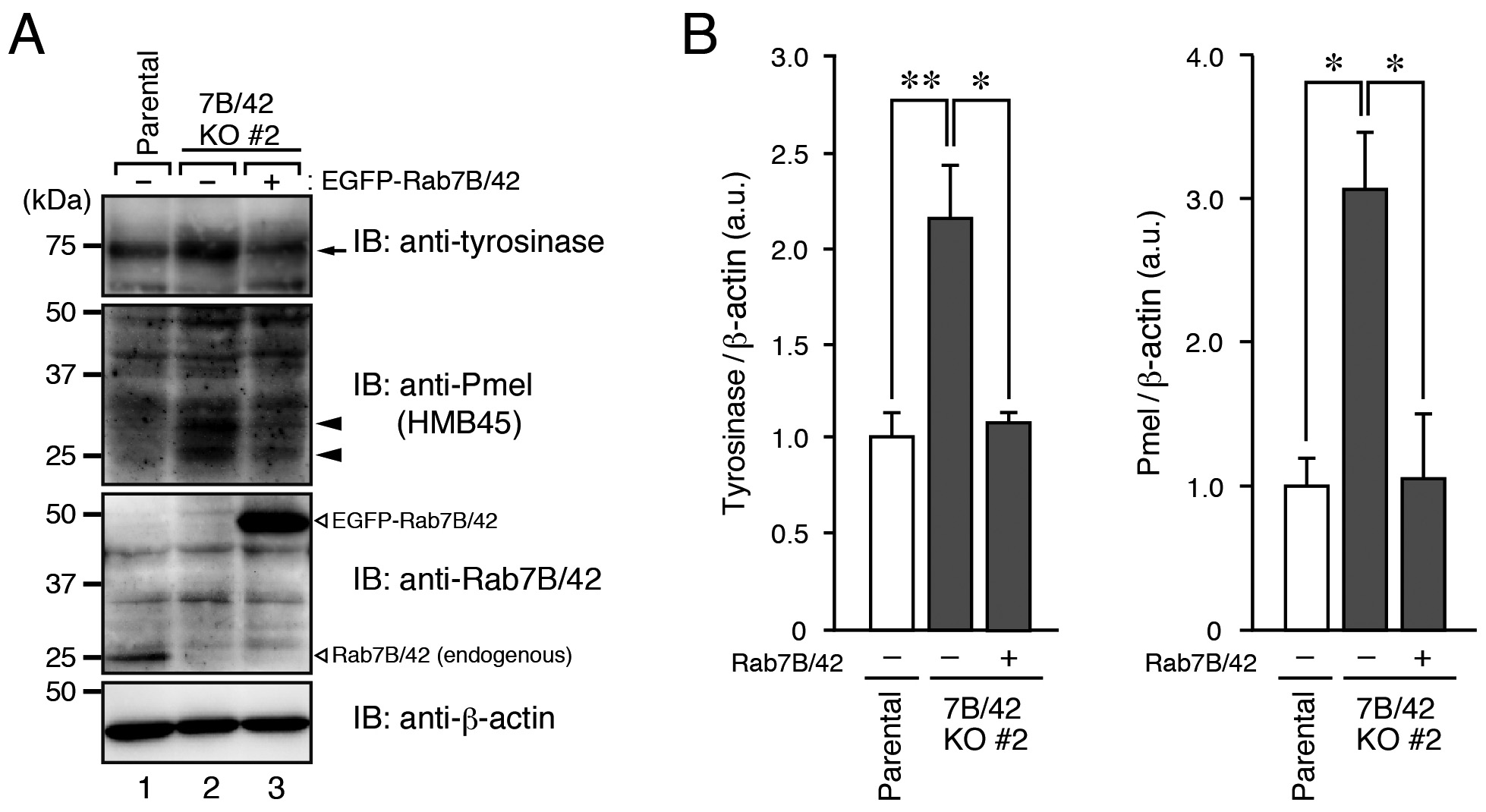

Supplement: Supplementary file 4 — Supplemental Fig. S4 Inhibition of melanosomal protein degradation in another independent Rab7B/42-KO#2 clone. (A) Parental, Rab7B/42-KO#2, and Rab7B/42-KO#2 cells stably expressing EGFP-Rab7B/42 were incubated for 36 hours with melanosomes. The lysates of the melanosome-containing compartment or the total cell lysates (for Rab7B/42 and β-actin) were analyzed by immunoblotting with the antibodies indicated. Two higher bands of Pmel (~37 kDa and ~50 kDa; see Fig. 6A) were not evident, presumably because the amount of cell lysates loaded in Fig. S4A was smaller than that in Fig. 6A. (B) Quantification of tyrosinase (arrow) and Pmel bands (arrowheads) shown in the top and second panels, respectively, in A (mean+s.e.m.; n=3). *, p<0.05; **, p<0.01 (Tukey’s test). [file csf_45_19039_4.jpg]

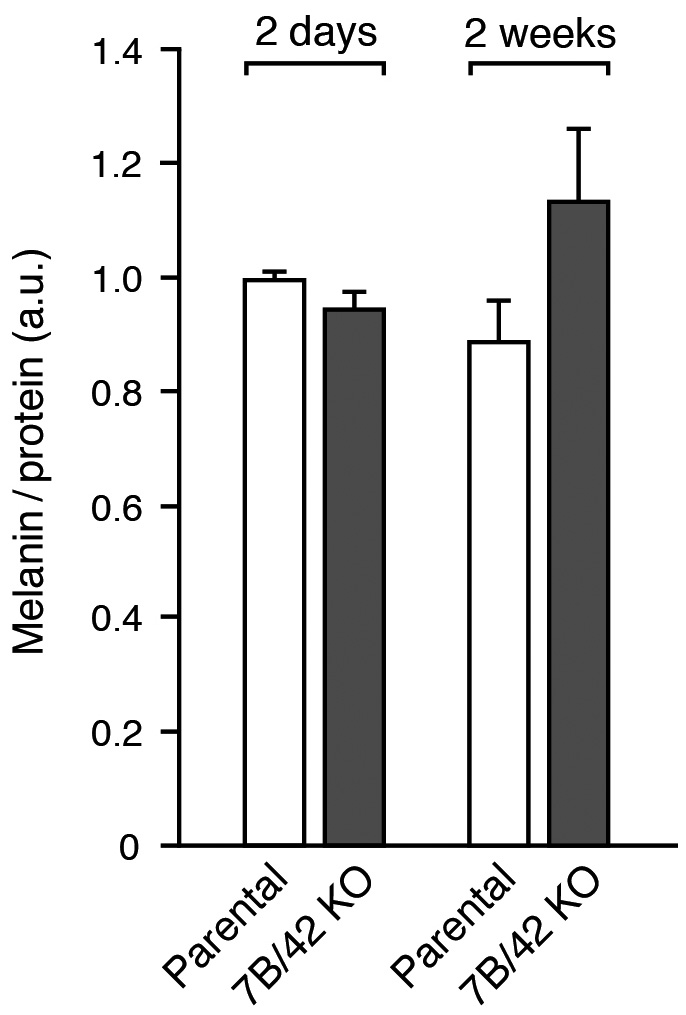

Supplement: Supplementary file 5 — Supplemental Fig. S5 Melanin content of keratinocytes after melanosome uptake (related to Fig. 5). Parental and Rab7B/42-KO cells were incubated for 48 hours with melanosomes and then cultured for two weeks in the absence of melanosomes. Their melanin content was measured as optical density. The bars represent the means+s.e.m. of the data obtained in three independent experiments. [file csf_45_19039_5.jpg]
